# Effect of Taking a Break From Cochlear-Implant Use for Resolving Facial-Nerve Stimulation: A Case Series

**DOI:** 10.1097/MAO.0000000000004698

**Published:** 2025-12-22

**Authors:** Marina Salorio-Corbetto, Susan T. Eitutis, Yu Chuen Tam, Robert P. Carlyon, John Deeks, Deborah Vickers, Zebunnisa H. Vanat, Dakota Bysouth-Young, Nicola Clarke, Muhammed Ayas, Charlotte Garcia, Matthew E. Smith, Manohar L. Bance

**Affiliations:** a Cambridge University Hospitals, Emmeline Centre for Hearing Implants, Cambridge, UK; b Cambridge Hearing Group, Cambridge, UK; c SOUND Lab, Department of Clinical Neurosciences, University of Cambridge, Cambridge, UK; d MRC Cognition & Brain Sciences Unit, University of Cambridge, Cambridge, UK; e SENSE Lab, Department of Clinical Neurosciences, University of Cambridge, Cambridge, UK

**Keywords:** Cochlear implants, Facial nerve stimulation, Nonauditory stimulation, Overstimulation

## Abstract

**Objective:**

To determine if a break from cochlear-implant (CI) stimulation reduces auditory electrical stimulation levels, to manage secondary facial nerve stimulation (FNS).

**Study design:**

Retrospective case review.

**Setting:**

Multidisciplinary clinic within a CI tertiary-care center.

**Patients:**

Four adult CI users exhibiting FNS at stimulation levels needed for auditory perception.

**Intervention:**

Taking a break from CI stimulation (temporary nonuse) to test the hypothesis that FNS arose from increasing auditory stimulation. An increase in auditory stimulation levels may stem from a decrease in auditory-nerve excitability due to overstimulation. A break would allow recovery of excitability, leading to lower current use and less cross-stimulation of the facial nerve. Break duration ranged between 1 week to several months.

**Main outcome measures:**

Lower and upper electrical stimulation levels required for hearing (N=3), reported or observed FNS (N=4), and FNS thresholds (N=1) before and after the break.

**Results:**

In all cases for whom stimulation levels for hearing were measured both before and after a break (*Patients 1* to *3*), these decreased after the break. In 2 of these cases, the decreased stimulation levels helped to manage persistent FNS. In one other case (*Patient 4*), FNS thresholds decreased slightly or remained unchanged after the break and the stimulation of most electrodes continued to lead to FNS with no change in auditory perception.

**Conclusions:**

A break from CI stimulation could be considered to manage persistent FNS when auditory stimulation levels increase over time. In some cases, a break may reduce the stimulation levels required for hearing, minimizing or eliminating the occurrence of FNS. Further research is needed to determine the biological mechanisms involved, optimize the duration of the break, as well as determine its long-term effectiveness.

## Introduction

Electrical stimulation may induce facial movements (usually eyelid and/or lip) due to the spread of the intracochlear current injected by the CI to the facial-nerve (FN) canal. This is termed FNS, occurs at an overall rate of 5.3%^1^ and can first appear at CI switch or at months or years later.^1–5^ The number of electrodes generating FNS for a single patient can increase over weeks or even years.^4^

FNS is often associated with conditions affecting the electrical impedance of the otic capsule bone, leading to abnormal current pathways. Hence, it has been reported with otosclerosis,^1–4,6,7^ otosyphilis,^4,7^ osteoporosis,^7^ meningitis,^6–8^ temporal-bone fracture,^6,7^ preimplantation or postimplantation closed-head injury,^6^ and cochlear malformations.^1,6^ However, it is often present without any of these conditions.^6,7^ CT imaging of affected patients may show bone demineralization, or soft tissue continuity of the cochlear-FN canals due to trauma or dehiscence,^4^ but most commonly no such abnormalities are noted.^4,8^

Even without known anatomical or histological abnormalities, current spread to the FN canal may also arise if excessively high stimulation levels are required for auditory perception due to nerve hypoplasia,^3^ high intracochlear impedances, or a history of super-power hearing aids.^3^ As with acoustic overstimulation, electrical overstimulation may reduce the excitability of the auditory nerve. For acoustic stimulation, the underlying mechanism is primarily glutamate excitotoxicity,^9^ which is known to affect inner hair cell (IHC)-auditory nerve synapses.^9–14^ Glutamatergic projections exist ipsilaterally and contralaterally beyond the level of the IHC-auditory nerve synapse.^15^ Ototoxicity may occur at different sites depending on the amount of overstimulation. For instance, Schwitzer et al^16^ showed that, in guinea pigs, the electrically evoked compound action potential (eCAP) threshold (synchronous peripheral auditory nerve response) increases after stimulating at 60 current level (CL) units above the initial eCAP threshold for 8 hours, while the threshold for the electrically evoked auditory brainstem response (eABR, a measure of brainstem responses) remains unchanged. However, using a longer period of stimulation (90 days for 16 hours a day), Basta et al^17^ observed bilateral changes in cell density within the central auditory pathway of guinea pigs.

Accordingly, the rationale for our intervention was that, if auditory nerve overstimulation underpins the increase in electrical stimulation levels needed for auditory sensation, a break from stimulation might reduce these levels by allowing the auditory neural axis to recover.

**Figure 1:**
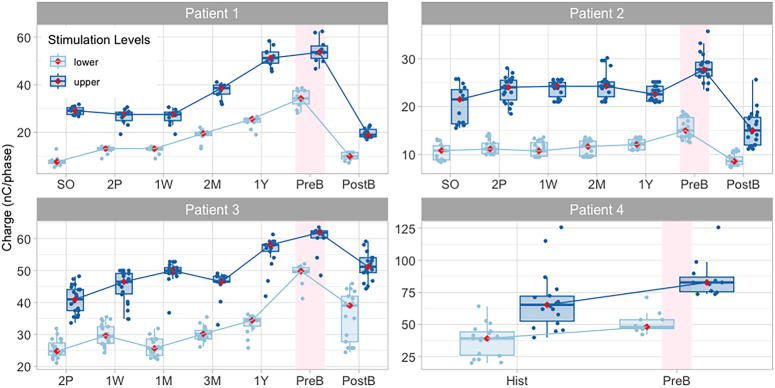
Lower and upper stimulation levels at different time points in the implantation pathway. Each dot represents a stimulation level for a given electrode. Boxplots represent the interquartile range; whiskers represent the range between the maximum and minimum values that are not considered outliers. Medians are represented by horizontal lines within each boxplot, but have also been marked with a red diamond. Medians for lower levels and upper stimulation levels measured along time are joined by lines to facilitate the visualisation of changes over time. The pink-shaded area highlights the stimulation levels measured at the prebreak appointment. 1M indicates 1 month after SO; 1W, 1 week after SO; 1Y, 1 year; 2M, 2 months after SO; 2P, second programming appointment; 3M, 3 months after SO; Hist, historical (early pathway data is not appropriate for comparison); PostB, first appointment after taking a break from stimulation; PreB, last appointment before taking a break from stimulation; SO, switch-on.

Here, we discuss 4 patients referred to our *H*earing *I*mplant *D*iagnostic and *O*ptimization (HIDO) clinic (Table 1), reviewed in the context of a service evaluation. For these patients, it was not possible to reduce or eliminate FNS by applying the treatment options currently available (changing the simulation parameters,^3,6,7,18^ deactivating electrodes, or limiting the upper stimulation levels below the FNS thresholds while maintaining audibility^4,6–8,18^). These patients persistently reported insufficient loudness despite their high levels of stimulation (Supplemental Table 1 and Supplemental Figure 1, Supplemental Digital Content 3, http://links.lww.com/MAO/C245), which had increased over time 3 of 4 cases (Fig. 1). Nine patients were offered a break to avoid nonuse, and possible explantation. One patient declined the break, and for 4 patients the data were incomplete, and therefore not reported in the main text.

**Table 1:** Etiology and relevant history of the patients

						No. electrodes leading to FNS	
Patient	Etiology and relevant history	CI brand and model	EC	FNS onset	Duration of break	Before break	Just after break
1	Maternal Rubella—bilateral hypoplasia of the vestibule and semi-circular canals	MED-EL CONCERTO FLEX28	2	5 y post switch-on	14 wks and 2 d	4	0
2	Measles	Cochlear^TM^ CI422	0	2 wk post switch-on	3 wk	12	0
3	Optic atrophy 1 (OPA1) mutation	Cochlear^TM^ CI24RE CA	0	Switch-on	Uncertain, up to 6 mo	No data available	No data available
4	Meningitis	Cochlear^TM^ CI22	0	13 years post switch-on	1 wk	16	16

CI model is described with brand and electrode type. Extracochlear electrodes (EC) due to incomplete insertion of the array are reported. The time between switch-on (SO) and FNS onset is given. The number of electrodes whose stimulation led to FNS before and after the break is specified.

In what follows, we will use the term “lower stimulation level” to refer to the T (Cochlear) or Thr (MED-EL) levels, and the term “upper stimulation level” to refer to C (Cochlear) or MCL (MED-EL). Stimulation levels, where available, were collected retrospectively from clinical maps. Supplemental Table 2, Supplemental Digital Content 3, http://links.lww.com/MAO/C245, gives the programming parameters of each map reported. Stimulation levels are reported in charge units (nC/phase). For MED-EL devices, charge units are specified in the programming software. For Cochlear devices, conversion formulae can be found in Supplemental Table 3, Supplemental Digital Content 3, available at http://links.lww.com/MAO/C245.

## Case series

### Patients for whom stimulation levels prebreak and postbreak were measured

**Figure 2:**
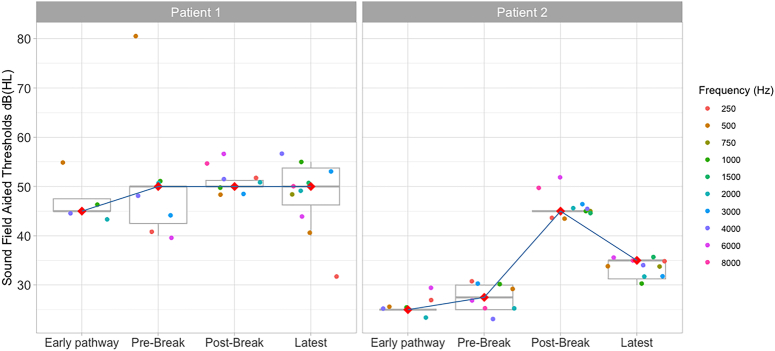
Boxplots showing aided sound field auditory thresholds (SFATs) at different time points for *Patients 1* and *2*. Note that median thresholds are indicated in red for each of the time points. Each dot represents a SFAT threshold for a given test frequency, indicated by the colour of the dot. Boxplots represent the interquartile range; whiskers represent the range between the maximum and minimum values that are not considered outliers. Medians are represented by horizontal lines within each boxplot, but have also been marked with a red diamond.

**Figure 3:**
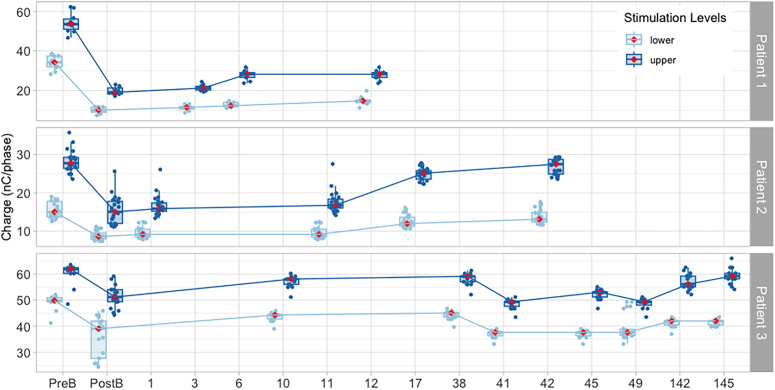
Long-term stimulation levels for 3 patients for whom long-term data are available postbreak. Thereafter, numbers represent months after Post-B. Note that the scale is not linear to facilitate visualization. Boxplots represent the interquartile range; whiskers represent the range between the maximum and minimum values that are not considered outliers. Medians are represented by horizontal lines within each boxplot, but have also been marked with a red diamond. Medians for lower levels and upper stimulation levels measured along time are joined by lines to facilitate the visualization of changes over time. PostB indicates first appointment after taking a break from stimulation; PreB, last appointment before taking a break from stimulation.

*Patient 1* had congenital hearing loss and a learning disability due to maternal rubella. They used a MED-EL CONCERTO FLEX28 CI and a bone-anchored hearing aid contralaterally (Table 1). The implanted ear had little auditory stimulation over 35 years preimplantation. Upon implantation, intense sounds led to pain. Voltage matrices suggested apical and mid-array current spread (Supplemental Figure 2, Supplemental Digital Content 3, http://links.lww.com/MAO/C245). The patient reported electric shock sensation (3 months post switch-on), headaches (1 year post switch-on), and FNS when wearing their processor (5 years post switch-on, after reporting hitting their head on the same side as the implant). A CT scan confirmed that the device remained inserted into the cochlea (albeit insertion was partial, with 2 extra-cochlear electrodes) and an integrity test did not show evidence of device failure. On referral, CI-alone sound field auditory thresholds (SFATs) were high, between 30 and 40 dB HL (Fig. 2), speech discrimination was poor [3/7 sounds in the “Auditory Speech Sound Evaluation” (A§E) phoneme discrimination test,^19^ see Table 2] and there was no detection of low-frequency sounds (such as /m/, /a/, /u/). Attempts were made to manage FNS by deactivating basal electrodes (11 to 12), increasing the pulse duration for apical electrodes (1 to 4) from 67.92 µs up to 150.2 µs, for medial electrodes (5 to 8) from 56.67 to 90.4 µs to 112.9 µs, and for the active basal electrode (10) from 45.42 to 75.42 µs, and modifying the loudness mapping function (*maplaw*=8000). Increasing the stimulation rate led to lower loudness when the rate was above 1000 pulses per second (pps). Triphasic stimulation provided similar patterns of FNS and loudness as biphasic pulses. Changing stimulation strategy (from FS4 to HDCIS), was also unsuccessful. After a 14-week break and then remapping, lower and upper stimulation levels dramatically decreased (Fig. 1). Records indicate that FNS re-occurred for 1 electrode (7), and pain for 2 other electrodes (9 and 10). The final map showed changes in pulse duration (from 27.08 to 50 µs) for several electrodes (6 to 7 and 9 to 10), with the absence of FNS or pain. In further follow-up visits (Fig. 3), lower and upper stimulation levels were increased to provide listening comfort or to improve sound detection (2.5 months and 6 months after the break), the loudness mapping function was modified (*maplaw* changed from 500 to 1000) to increase loudness for low-level sounds, and electrode 2 was deactivated due to poor SFAT response. Despite these changes, stimulation levels continued to be lower than before the break. CI-alone SFATs continued to be raised (Fig. 2), but were similar to the SFATs measured with their contralateral bone-anchored hearing aid. Further increases in upper stimulation levels were deliberately avoided to prevent recurrent FNS. The patient was able to use their CI consistently, together with their bone-anchored hearing aid. Bimodal performance for Bamford-Kowal-Bench (BKB) sentences^20^ with both devices was better compared with bone-anchored-hearing-aid alone (Table 2). Twenty-one months after the break, the patient described their CI as “their long-distance radar,” suggesting that it served as an aid to communication, sound awareness, and distant listening. As a barrier to obtaining accurate feedback from the patient was suspected, an individualized custom-made sound diary (Supplemental Digital Contents, Appendix 1, Supplemental Digital Content 1, http://links.lww.com/MAO/C243) was used after the break to help the patient reflect on their experiences and to obtain a record of symptoms of overstimulation.

**Table 2:** Summary of A§E and BKB outcomes

	A§E			BKB MQ (word score)		
Patient	Early pathway or earliest available outcome	Before break	After break	Early pathway or earliest available outcome	Before break	After break
1	0% (0/7, 3.5 mo before taking a break)	0% (2 mo before taking a break, 0/7)	29% (2/7, 6 mo after the end of the break)	Not performed	Not performed	6 mo postend of break: 0% CI only 73% bimodal 6 mo postbreak 96% bimodal 12 mo postbreak
2	43% (1 mo post switch-on, 3/7) 55% (5 mo post switch-on, 11/20)	60% (12/20, 4.5 mo before taking a break)	55% (11/20, 2 mo after the end of the break) 65% (13/20, 11 mo after the end of the break) 65% (13/20, 17 mo after the end of the break)	Not performed	Not performed	Not performed
4	100% (20/20, 7 y and 1 mo before taking a break)	95% (19/20, 5 mo and 1 wk before taking a break)	Not tested	95% (6 y and 7 mo before taking a break)	72% (5 mo and 1 wk before break)	Not repeated, no change in map

For BKB sentences the talker was a male in quiet (BKB MQ). No records are available for *Patient 3*.

*Patient 2* was diagnosed with hearing loss at 18 months of age after measles (Table 1). They wore a hearing aid unilaterally from age 4. At age 62, they received a hearing aid in the contralateral ear, and, at age 65, this ear was implanted with a Cochlear CI422. After implantation, they continued wearing a hearing aid in the nonimplanted ear. Insufficient loudness and compliance issues prompted increases in pulse duration from 37 to 50 µs within the first week after switch-on. FNS (blepharospasm) presented within the first 2 weeks of CI use. Subsequently, pulse duration increases up to 150 µs failed to eliminate FNS. One year after switch-on, data logging records indicated that the patient wore the CI for only 3.5 to 5 hours a day. Over the course of the next 3 years, appropriate loudness could not be achieved without triggering FNS (blepharospasm and hemifacial spasms). In addition, the patient reported headaches and tinnitus, present only with processor use. Normal transimpedance matrices (TIM) suggested no significant current shunting out of the cochlea (Supplemental Figure 3, Supplemental Digital Content 3, http://links.lww.com/MAO/C245). A CT scan ruled out electrode migration. After a 3-week break, upper and lower stimulation levels decreased (Fig. 1), and no FNS was observed using a pulse duration of 50 µs. However, increases of the lower and upper stimulation levels were needed at 1 and 9 moths postbreak due to insufficient loudness (Fig. 3). FNS recurred nine months after the break (eye twitch) and the patient reported not hearing as well as before. Consequently, increases were made to the stimulation levels (Fig. 3). Performance (Table 2) and device wearing time remained limited (average usage: 3.6 h/d before the break vs. 3.7 h after the break).

*Patient 3* had hearing loss associated to dominant optic atrophy due to OPA1 mutation (Table 1). Hearing loss was diagnosed at 6 years of age, but they were implanted in their adulthood with an Advanced Bionics device. Within the course of 1 year after implantation, they reported intermittent sound, pain, and facial twitching, poor battery life (2 to 3 h), and “electric shock,” and thus they were reimplanted. Their second device was a Cochlear CI24RE CA device. After reimplantation, loudness was low despite high levels of stimulation, the dynamic range was narrow, and severe FNS and pain occurred. Sound quality was described as “beeping” or “buzzing.” Audibility decreased over time. Parallel deterioration in both vision and balance was reported. An integrity test did not demonstrate device failure. Mapping modifications, including increases in pulse duration up to 200 µs, changes in stimulation rate, disabling of electrodes, and using different modes or stimulation (monopolar 1+2, monopolar 2, bipolar) were unsuccessful. An auditory brainstem implant was offered, but the patient declined. The patient reported several periods of nonuse due to a lack of sound perception and pain or FNS. After each of these breaks, the patient was able to hear again with their processor on, but eventually loudness would lessen and the pain would become unbearable, leading to another period of nonuse. Here, we report one instance documented in the programming notes, where the patient spontaneously took a “long” break from stimulation between 2 sessions, separated by 2 months. After the break, stimulation levels decreased (Fig. 1). Two months later, after consistently using the device, the stimulation levels rose, albeit without reaching the prebreaks levels. Ten-year follow-up data indicates that stimulation levels remained high and tended to increase over time, even after inconsistent use of the device (Fig. 3). Loudness and clarity continue to be insufficient, and the issues with pain and FNS persist.

### A patient for whom FNS thresholds were measured before and after a break

**Figure 4:**
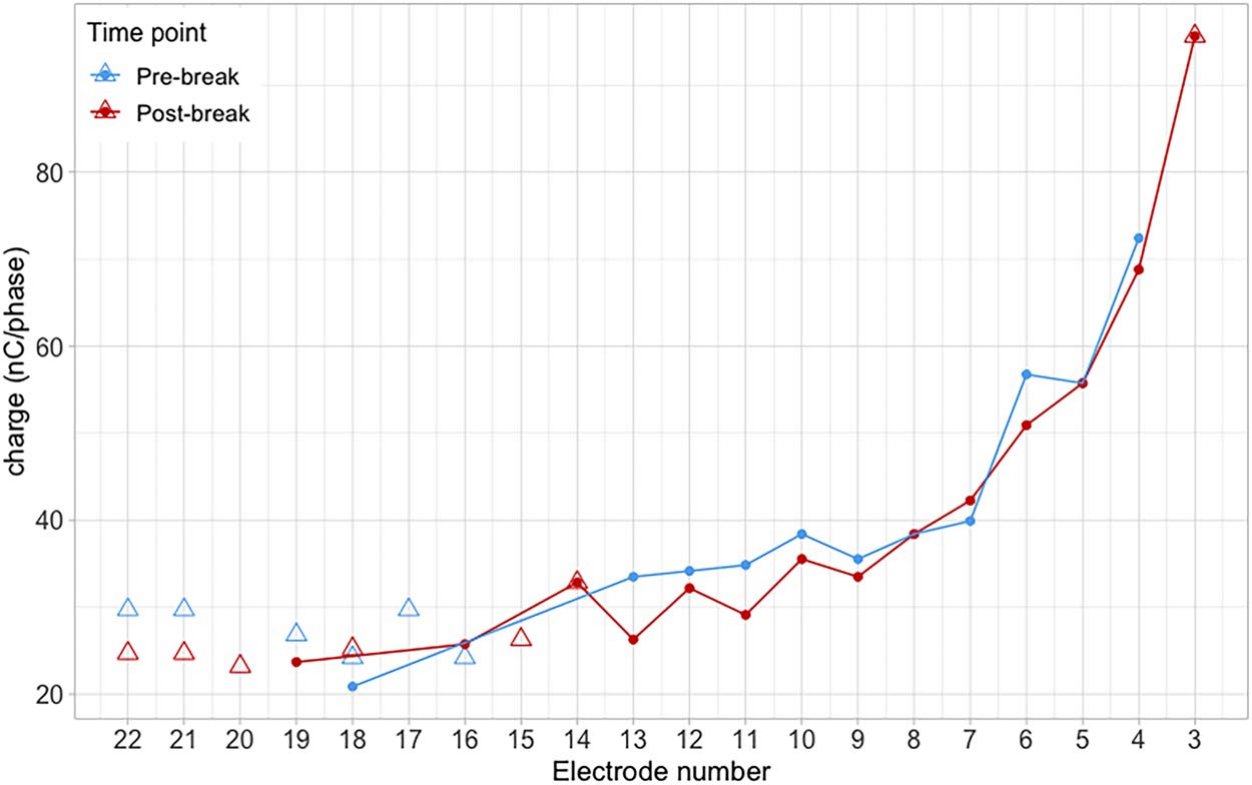
Facial-nerve stimulation thresholds (points) before the break (blue) and after the break (red) for *Patient 4*. Charge levels corresponding to a loudness rating of “loud” are denoted by triangles and were obtained at some electrodes only before the break (blue) and after the break (red). All measurements were carried out using a Pseudomonopolar grounding mode, a 150 µs pulse duration, and a pulse rate of 210 to 245 pps.

*Patient 4* had a history of meningitis. They received a CI at age 48 years (Cochlear CI22M). Pain was reported 10 years after switch-on and FNS 3 years later (Table 1). Increasing pulse duration up to 150 µs overall, and up to 400 µs for FNS-inducing electrodes, trialling different grounding configurations (Common Ground [CG], Bipolar+3, and Pseudomonopolar), and deactivating up to 9 electrodes, failed to reduce FNS and significantly reduced auditory performance based on subjective report. Despite substantial programming modifications, BKB scores failed to return to pre-FNS levels (Table 2). FNS was severe, manifesting as blepharospasm, pain, and “electric shock” that, once activated by a period of CI use, persisted even without the processor switched on, although it was much worse when the processor was worn and switched on. After a 1-week break, FNS thresholds decreased slightly for some electrodes and remained unchanged for other electrodes (Fig. 4). Unfortunately, there are no recorded auditory lower stimulation levels after the break. Limited data are available for 3 electrodes for which the charge levels required to elicit a “loud” rating were recorded. Levels decreased slightly for 2 electrodes and kept the same for one. For most electrodes, it was not possible to obtain a “comfortably loud” rating below the FNS threshold. No programming changes were deemed necessary and the patient continued to use the same map as before the break. It is noteworthy that, for this case, available records for the 5 years before the break indicate that programming levels remained relatively stable, unlike for the other cases described here (Fig. 1). This patient was treated by reimplantation with an Oticon Medical device that delivered pseudo-monophasic pulses and returned 80% of the current inside the cochlea. Reimplantation outcomes, but not the effect of taking a break, have been previously reported by our group.^5^

## Discussion

The present report raises the issue of potentially overstimulating the auditory nerve using clinical maps. For patients whose stimulation levels for auditory perception were measured before and after the break (*Patients 1*, *2*, and *3*), there was a postbreak reduction in stimulation levels. This is illustrated in Figure 1. Changes in stimulation levels can be expressed as a percentage of the prebreak dynamic range^21^ to determine whether they are meaningful. Lower stimulation levels decreased by 121%, 51%, and 123% and upper stimulation levels decreased by 173%, 101%, and 79% for *Patients 1*, *2*, and *3*, respectively. These percentages are well above the criterion of 20% suggested by Gajadeera et al^21^ for stable stimulation levels across consecutive sessions. Full details about these calculations are shown in the Supplemental Table 4 (Supplemental Digital Content 3, http://links.lww.com/MAO/C245). For the one other patient whose FNS thresholds were recorded (*Patient 4*), these remained unchanged after the break and no programming changes were made, suggesting that stimulation levels remained relatively stable immediately after the break. The persistence of FNS and the little change in reported loudness suggest that the break was not effective for this patient.

It is not possible, from our current data, to establish whether excitotoxicity was a mechanism mediating the increase in stimulation levels and the subsequent decrease experienced by these patients after the break. However, based on recent publications suggesting that electrical stimulation could lead to excitotoxic effects,^17,22^ we propose that clinicians should proceed with caution when patients show a progressive increase in their stimulation levels, especially if the increase affects both lower and upper levels of stimulation. For children, both lower and upper levels of stimulation may initially change,^23,24^ but they should reach stability by the first-year visit.^24^ Patients showing changes in stimulation levels >20% of the dynamic range after 3 months postimplantation should be monitored.^21^ Appropriate counselling should be used to ensure that patients are reporting on loudness and not on clarity.

It is helpful for clinicians to consider the possible reasons for the increase in stimulation levels over time. Some prelingually deaf patients may find it hard to report on loudness when determining upper stimulation levels. In addition, some patients using bimodal stimulation may try to “match” the sound quality of the 2 devices, which provide markedly different sensations. This can lead to an overestimation of the upper stimulation levels. Consistent with this, *Patient 1* and *Patient 2* reported headaches that occurred only with the device on. Lower stimulation levels may also be overestimated in some cases, which would lead to the intrusive perception of low-level sounds. Consistent with this, *Patient 2* reported “tinnitus” with the CI only. In other cases, high stimulation levels may be needed due to tissue growth or fibrosis developing after implantation.^25^ This should, theoretically, be accompanied by changes in contact impedances if the growth is large enough. We would not expect cases like these to benefit from taking a break.

Another relevant point of discussion is the long-term duration of any effects of taking a break from stimulation. For 2 of the cases reported (*Patients 1* and 2), stimulation levels remained below prebreak levels in follow-up sessions, and FNS was decreased or eliminated. However, for *Patient 3*, stimulation levels tended to increase over time, and fluctuations in loudness, pain, and FNS persisted.

It is of interest to reflect on the clinical features that may be associated with a lack of benefit from a break from stimulation. Among the 4 cases reported here, a break was not sufficient in managing FNS for 2 out of 4 patients. One of these patients, *Patient 3*, had OPA1 mutation, a progressive condition characterised with demyelination and axonal loss in its advanced stage.^26^ Due to the abnormalities of the auditory nerve, if excitotoxicity played a role in increasing the stimulation levels, a break might have decreased these, but would not necessarily be expected to improve sound quality or intelligibility. In addition, changes over time may reflect disease progression. The other patient who did not benefit from a break was *Patient 4*, who had stable maps for the last 5 years before this report and had a history of meningitis. Patients with meningitis are known to require high levels of electrical stimulation to achieve auditory perception,^27^ especially if ossification is present. It is possible that in this case the high levels required were not driven by overstimulation.

This report has several limitations. First, as the programming levels documented here were obtained retrospectively, there were some differences in pulse rate and pulse duration across the compared maps. Upper and, more so, lower stimulation levels tend to decrease with increasing stimulation rate,^28,29^ with rate-threshold slopes being steeper for rates above 1000 pps.^29^ These effects are variable across individuals, with the slopes of the lower stimulation level versus rate function below 1000 pps thought to reflect some aspect of neural health.^29–31^ For *Patients 1* and *2*, stimulation levels were measured using different pulse rates before and after the break (Supplemental Table 2, Supplemental Digital Content 3, http://links.lww.com/MAO/C245). While for *Patient 2* pulse rate was lower after the break than before the break (500 pps prebreak vs. 250 pps postbreak), the opposite was true for *Patient 1* (518 pps prebreak vs. 1205 pps postbreak). It could be argued that the decrease in the stimulation levels for *Patient 1* is at least partially due to the faster pulse rate used to measure the postbreak thresholds. However, the size of postbreak changes in both the lower and upper levels was substantially larger than has been reported for changes in pulse rate over this range, making this explanation highly unlikely.^32,33^ Another issue is that the amount of charge required for the threshold is not constant across pulse durations.^28^ This complicates the comparison of stimulation levels measured with different pulse durations. For *Patients 1* and *2*, pulse duration was generally longer before than after the break (Supplemental Table 3, Supplemental Digital Content 3, http://links.lww.com/MAO/C245). This is consistent with the usual clinical practice of increasing pulse duration to manage FNS, which was no longer needed after the break. It is difficult to estimate how this might have affected individual patients, as the effect of pulse duration on thresholds depends on physiologically vulnerable charge-integration mechanisms and thus varies across listeners.^34^ Furthermore, recorded programming levels are affected by standard programming procedures such as loudness balancing of upper levels, and may be influenced by clinical decision processes. For example, for *Patients 1* and *2*, increases of the upper stimulation levels were deliberately avoided to prevent FNS. It is also necessary to consider that patients may have learned over time to provide more accurate responses in psychophysical measurements. This could have, at least partially, decreased the stimulation levels without a change in excitability. Finally, breaks varied in duration across patients. A systematic approach, including subjective and objective measures, and similar break durations, is needed to investigate the effect of taking a break from stimulation on the excitability of the auditory nerve and to identify any factors underpinning differences in the effects of a break across patients. Monitoring progress with functional testing and electrophysiological measures is likely to provide contextual information to interpret any changes in current requirements and any post-break effects. Based on recent findings on excitotoxicity,^16,17^ eCAP and the eABR could be useful in capturing any excitotoxic effects on auditory responses. In addition, the electrically evoked Auditory Steady State Response (eASSR)^35^ or cortical onset responses (CORs or CAEPs)^36,37^ may provide objective information about loudness growth. To aid monitoring in the future, we have designed a template that can be found in Appendix 2, Supplemental Digital Content 2, http://links.lww.com/MAO/C244.

## Sources of funding

M.S.-C. has received research funding from Cochlear Corporation, Oticon Medical, and MED-EL. S.E. has received funding from Advanced Bionics. D.B.-Y. has received funding by Cochlear Corporation. D.V. was funded by an MRC Senior Fellowship in Hearing (MR/S002537/1). D.V. and M.B. received funding from the Cambridge NIHR Biomedical Research Centre [NIHR203312]. The views expressed are those of the authors and not necessarily those of the NIHR or the Department of Health and Social Care.

## Supplementary Material

SUPPLEMENTARY MATERIAL
