# Supplementary material for: Effect of Taking a Break From Cochlear-Implant Use for Resolving Facial-Nerve Stimulation: A Case Series
Source: Otol Neurotol. 2025 Dec 22;47(2):e193–200. doi: 10.1097/MAO.0000000000004698 (PMC12777617; doi:10.1097/MAO.0000000000004698)
Supplement: SUPPLEMENTARY MATERIAL [file mao-47-e193-s001.docx]

The following example of a sound diary was designed for an individual considering their needs and the key relevant questions required to support them. Its inclusion here is meant as an model to aid the development of similar customised tools. This diary is not to be re-utilised for all cases in its current version. Instead, it is suggested that sound diaries are designed for each case to ensure that they are accessible to the user and include questions that are relevant to their case.

1. **Did you feel any pain today around your eye or head?**

**No Unsure Yes Yes, too much**
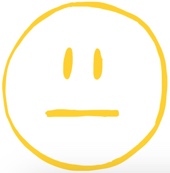

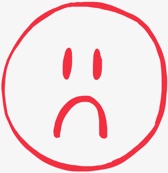

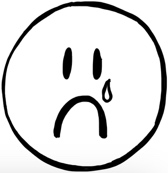


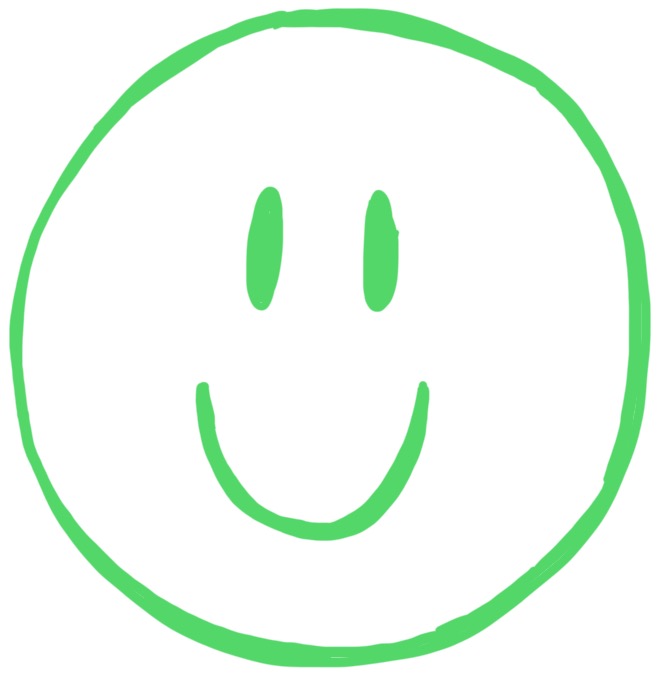


1. **Did your eye twitch today when you were wearing your implant?**

**No Unsure Yes Yes, too much**
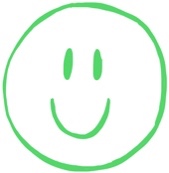

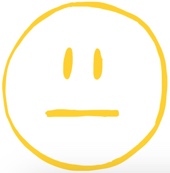

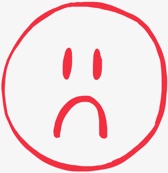

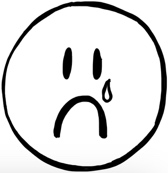


1. **Did you hear any tapping sounds when your implant was off?**

**No Unsure Yes Yes, too much**
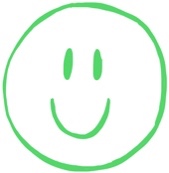

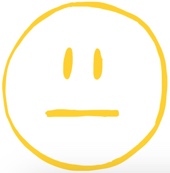

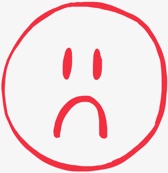

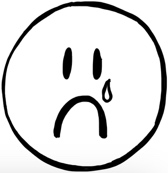


1. **Did you hear any tapping sounds when your implant was on?**

**No Unsure Yes Yes, too much**
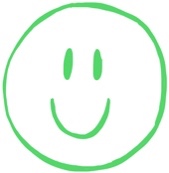

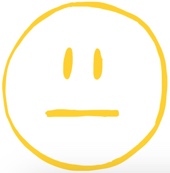

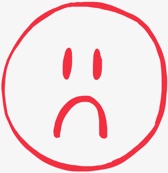

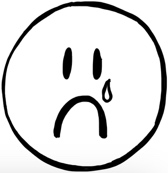


1. **Could you hear any other sounds that were not just tapping today?**

**No Unsure Yes**

**
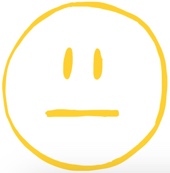

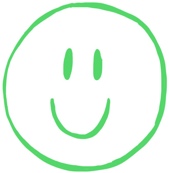
**


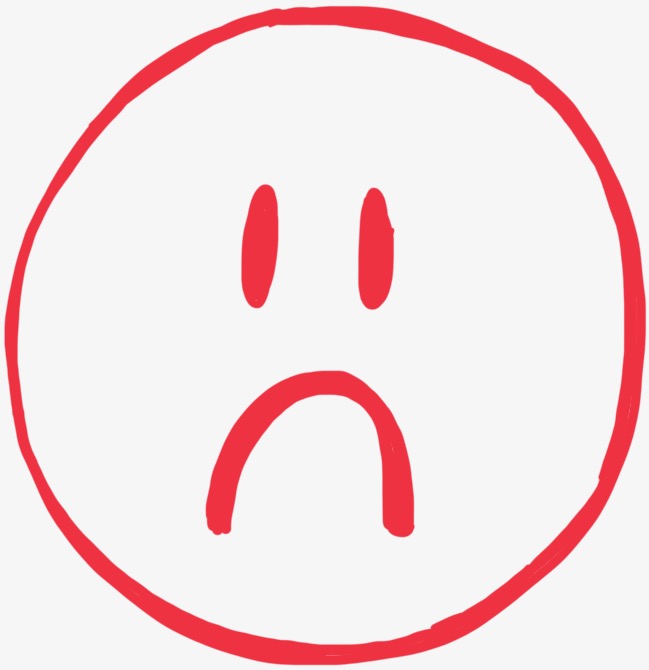


1. **Could you hear people talking and their speech with your implant today?**

**No Unsure Yes**


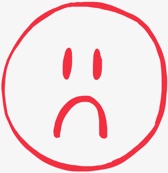

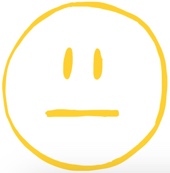

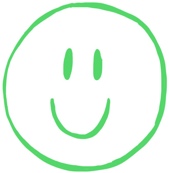


1. **Do you feel your implant has helped your hearing today?**

**No Unsure Yes**


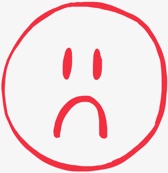
 **
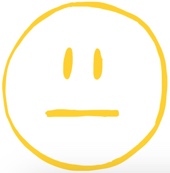

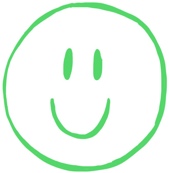
**

1. **Circle what activities you did today…**

| **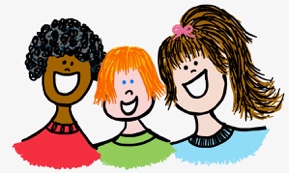**  **I spent time with friends or family** | **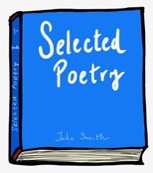**  **I read a book** | **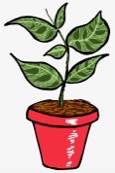**  **I did some gardening** |
| --- | --- | --- |
| **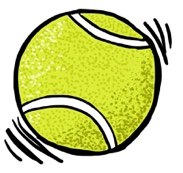**  **I practised sports** | **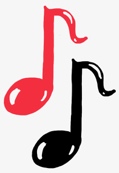**  **I listened to music** | **Other: _______________** |
