# Supplementary material for: Effect of Taking a Break From Cochlear-Implant Use for Resolving Facial-Nerve Stimulation: A Case Series
Source: Otol Neurotol. 2025 Dec 22;47(2):e193–200. doi: 10.1097/MAO.0000000000004698 (PMC12777617; doi:10.1097/MAO.0000000000004698)
Supplement: SUPPLEMENTARY MATERIAL [file mao-47-e193-s002.docx]

**FACIAL NERVE STIMULATION PROGRAMMING CHANGES CHECKLIST**

**Patient details**

Name

Date of birth

Hospital number

**Relevant history**

Aetiology of hearing loss:

Duration of deafness^1^ – Ask the patient:

- Before you had your cochlear implant, how long was your hearing at the same level as just before the implant?

- When did your first have trouble to listen through the phone?

Age at implantation:

**Cochlear-implant details**

Side: R/L Electrode type Processor model

**Bilateral aspects**

Bilateral user Y/N

Bimodal user Y/N

**Hearing-aid history for the implanted ear**

Previously aided? Y/N

Type of device

Duration of use

**Measurements to be completed**

All patients with FNS should have measurements for programming levels, FNS thresholds, eCAPs and eABR completed where possible. Measurements should be completed for patients who are experiencing worsening symptoms or who are not effectively managed with modifications to the clinical maps.

**Section 1 – Programming level history (before break)**

Fill in the table with levels converted into charge in order to assess whether programming levels increased over time. Formulae to express programming levels in nC/phase can be obtained from manufacturers. Patients showing an increase in programming levels > 20% relative to the dynamic range of the previous session^2^ after the third month session should be monitored.

| **Time point** | **Median T/Thr level** | **Median M/C level or MCL** | **Percentage change relative to the previous session DR** |
| --- | --- | --- | --- |
| First programming |  |  |  |
| Second programming |  |  |  |
| One month |  |  |  |
| Three months |  |  |  |
| One year |  |  |  |
| At referral |  |  |  |

**** please continue to section 2**

**Section 2 – Facial Nerve Stimulation Record (before break)**

- Obtain FNS thresholds for each electrode. These levels will be used for later comparison if a break is suggested and will not necessarily be used in a take-home map. Please select a mode of stimulation, pulse rate, and pulse duration and use them throughout testing. Investigations should not exceed M, C, or MCL (as applicable). If no FNS occurs at this level, change to the next electrode or end testing if all other electrodes have been investigated.

| Keep these settings constant throughout testing | Mode of stimulation | |  | |
| --- | --- | --- | --- | --- |
|  | Pulse rate | |  | |
|  | Pulse duration | |  | |
| Electrode number | T/Thr level | FNS threshold (please note M, C, or MCL and “NFNS” if no FNS is present at MCL) | Loudness rating at FNS (Employ a user-appropriate loudness scale and document; make sure you record descriptors, i.e. ‘loud’ and not just numbers). | Description of FNS (location and whether it is reported by the patient or visible to the examiner) |
| 1 |  |  |  |  |
| 2 |  |  |  |  |
| 3 |  |  |  |  |
| 4 |  |  |  |  |
| 5 |  |  |  |  |
| 6 |  |  |  |  |
| 7 |  |  |  |  |
| 8 |  |  |  |  |
| 9 |  |  |  |  |
| 10 |  |  |  |  |
| 11 |  |  |  |  |
| 12 |  |  |  |  |
| 13 |  |  |  |  |
| 14 |  |  |  |  |
| 15 |  |  |  |  |
| 16 |  |  |  |  |
| 17 |  |  |  |  |
| 18 |  |  |  |  |
| 19 |  |  |  |  |
| 20 |  |  |  |  |
| 21 |  |  |  |  |
| 22 |  |  |  |  |

**** please continue to section 3 for MAP changes and to section 4B/C/D for objective measures baselines**

**Section 3 – Optimising programming parameters**

1. **Effect of changing pulse duration**

If available for the patient’s implant, select a few electrodes that led to FNS in the previous step, and re-test the above using different pulse durations. Include electrodes along the whole array if possible.

| Keep these settings constant throughout testing | Pulse rate |  | | | |
| --- | --- | --- | --- | --- | --- |
|  | Mode of stimulation |  | | | |
| Electrode number | Pulse duration | T/Thr level | FNS Threshold (please note M, C, or MCL and NFNS if no FNS is present at MCL) | Loudness rating at FNS (Employ a user-appropriate loudness scale and document; make sure you record descriptors, i.e. ‘loud’ and not just numbers). | Description of FNS (location and whether it is reported by the patient or visible to the examiner) |
|  |  |  |  |  |  |
|  |  |  |  |  |  |
|  |  |  |  |  |  |
|  |  |  |  |  |  |
|  |  |  |  |  |  |
|  |  |  |  |  |  |
|  |  |  |  |  |  |
|  |  |  |  |  |  |
|  |  |  |  |  |  |
|  |  |  |  |  |  |
|  |  |  |  |  |  |
|  |  |  |  |  |  |

1. **Effect of changing mode of stimulation**

If available for the patient’s implant, select a few electrodes that led to FNS in the previous step, and re-test the above using different modes of stimulation. Include electrodes along the whole array if possible.

| Keep these settings constant throughout testing | Pulse rate |  | | | |
| --- | --- | --- | --- | --- | --- |
|  | Pulse duration |  | | | |
| Electrode number | Mode of stimulation | T/Thr level | FNS  Threshold (please note M, C, or MCL and NFNS if no FNS is present at MCL) | Loudness rating at FNS | Description of FNS (location and whether it is reported by the patient or visible to the examiner) |
|  | MP1 |  |  |  |  |
|  | MP2 |  |  |  |  |
|  | MP1+2 |  |  |  |  |
|  | BP+3 |  |  |  |  |
|  | CG |  |  |  |  |
|  | Pseudomonopolar |  |  |  |  |
|  | MP1 |  |  |  |  |
|  | MP2 |  |  |  |  |
|  | MP1+2 |  |  |  |  |
|  | BP+3 |  |  |  |  |
|  | CG |  |  |  |  |
|  | Pseudomonopolar |  |  |  |  |
|  | MP1 |  |  |  |  |
|  | MP2 |  |  |  |  |
|  | MP1+2 |  |  |  |  |
|  | BP+3 |  |  |  |  |
|  | CG |  |  |  |  |
|  | Pseudomonopolar |  |  |  |  |
|  | MP1 |  |  |  |  |
|  | MP2 |  |  |  |  |
|  | MP1+2 |  |  |  |  |
|  | BP+3 |  |  |  |  |
|  | CG |  |  |  |  |
|  | Pseudomonopolar |  |  |  |  |

**c - Effect of changing pulse shape**

If available for the patient’s implant, select a few electrodes that led to FNS in the previous step, and re-test the above using different pulse shapes. Include electrodes along the whole array if possible.

| Keep these settings constant throughout testing | Pulse rate |  | | | |
| --- | --- | --- | --- | --- | --- |
|  | Pulse duration |  | | | |
| Electrode number | Mode of stimulation | T/Thr level | FNS Threshold (please note M, C, or MCL and NFNS if no FNS is present at MCL) | Loudness rating at FNS | Description of FNS (location and whether it is reported by the patient or visible to the examiner) |
|  | Biphasic |  |  |  |  |
|  | Triphasic |  |  |  |  |
|  | Pseudomonophasic |  |  |  |  |
|  | Biphasic |  |  |  |  |
|  | Triphasic |  |  |  |  |
|  | Pseudomonophasic |  |  |  |  |
|  | Biphasic |  |  |  |  |
|  | Triphasic |  |  |  |  |
|  | Pseudomonophasic |  |  |  |  |
|  | Biphasic |  |  |  |  |
|  | Triphasic |  |  |  |  |
|  | Pseudomonophasic |  |  |  |  |

**Section 4 – Effect of taking a break from stimulation**

1. **Stimulation levels**

Measure lower and upper stimulation levels for at least some electrodes along the array

| Duration and dates of the break |  | | | | |
| --- | --- | --- | --- | --- | --- |
| Keep these settings constant throughout testing | Pulse rate |  | | | |
|  | Pulse duration |  | | | |
|  | Mode of stimulation |  | | | |
| Electrode number | Before the break  MAP number: | | | After the break  MAP number: | |
|  | T/Thr | | M, C, or MCL | T/Thr | M, C, or MCL |
| 1 |  | |  |  |  |
| 2 |  | |  |  |  |
| 3 |  | |  |  |  |
| 4 |  | |  |  |  |
| 5 |  | |  |  |  |
| 6 |  | |  |  |  |
| 7 |  | |  |  |  |
| 8 |  | |  |  |  |
| 9 |  | |  |  |  |
| 10 |  | |  |  |  |
| 11 |  | |  |  |  |
| 12 |  | |  |  |  |
| 13 |  | |  |  |  |
| 14 |  | |  |  |  |
| 15 |  | |  |  |  |
| 16 |  | |  |  |  |
| 17 |  | |  |  |  |
| 18 |  | |  |  |  |
| 19 |  | |  |  |  |
| 20 |  | |  |  |  |
| 21 |  | |  |  |  |
| 22 |  | |  |  |  |

1. **Facial nerve stimulation**

| Duration and dates of the break |  | | | | | |
| --- | --- | --- | --- | --- | --- | --- |
| Keep these settings constant throughout testing | Pulse rate | |  | | | |
|  | Pulse duration | |  | | | |
|  | Mode of stimulation | |  | | | |
| Electrode number | Before the break  (please note that you can transfer here the values from section 2 / 3a/ 3b / 3c as long as these thresholds are re-measured after the break using the same stimulation parameters) | | | After the break | | |
|  | FNS threshold | Loudness rating | Description  (location and whether it is reported by the patient or visible to the examiner) | FNS threshold | Loudness rating | Description  (location and whether it is reported by the patient or visible to the examiner) |
| 1 |  |  |  |  |  |  |
| 2 |  |  |  |  |  |  |
| 3 |  |  |  |  |  |  |
| 4 |  |  |  |  |  |  |
| 5 |  |  |  |  |  |  |
| 6 |  |  |  |  |  |  |
| 7 |  |  |  |  |  |  |
| 8 |  |  |  |  |  |  |
| 9 |  |  |  |  |  |  |
| 10 |  |  |  |  |  |  |
| 11 |  |  |  |  |  |  |
| 12 |  |  |  |  |  |  |
| 13 |  |  |  |  |  |  |
| 14 |  |  |  |  |  |  |
| 15 |  |  |  |  |  |  |
| 16 |  |  |  |  |  |  |
| 17 |  |  |  |  |  |  |
| 18 |  |  |  |  |  |  |
| 19 |  |  |  |  |  |  |
| 20 |  |  |  |  |  |  |
| 21 |  |  |  |  |  |  |
| 22 |  |  |  |  |  |  |

1. **Electrophysiological measures**

Keep the recording parameters the same across sessions.

**eCAP threshold**

| Duration and dates of the break |  | | | |
| --- | --- | --- | --- | --- |
| Keep these settings constant throughout testing | Probe rate |  | | |
|  | Pulse duration |  | | |
|  | Offset |  | | |
|  | Gain |  | | |
|  | Delay |  | | |
|  | Artefact reduction technique |  | | |
|  | Masker relative level |  | | |
|  | Interphase gap |  | | |
|  | Probe indifferent electrode |  | | |
|  | Masker indifferent electrode |  | | |
| Electrode number | Before the break  Visit date: | | After the break  Visit date: | |
|  | eCAP threshold | AGF slope | eCAP threshold | AGF slope |
| 1 |  |  |  |  |
| 2 |  |  |  |  |
| 3 |  |  |  |  |
| 4 |  |  |  |  |
| 5 |  |  |  |  |
| 6 |  |  |  |  |
| 7 |  |  |  |  |
| 8 |  |  |  |  |
| 9 |  |  |  |  |
| 10 |  |  |  |  |
| 11 |  |  |  |  |
| 12 |  |  |  |  |
| 13 |  |  |  |  |
| 14 |  |  |  |  |
| 15 |  |  |  |  |
| 16 |  |  |  |  |
| 17 |  |  |  |  |
| 18 |  |  |  |  |
| 19 |  |  |  |  |
| 20 |  |  |  |  |
| 21 |  |  |  |  |
| 22 |  |  |  |  |

**eABR threshold**

| Duration and dates of the break |  | | | |
| --- | --- | --- | --- | --- |
| Keep these settings constant throughout testing | Mode of stimulation |  | | |
|  | Probe rate |  | | |
|  | Pulse duration |  | | |
|  | Electrode montage | Positive |  | |
|  |  | Negative |  | |
|  |  | Ground |  | |
|  | Number of sweeps |  | | |
|  | Recording time window |  | | |
|  | Artefact reduction technique |  | | |
|  | Low pass filter cutoff |  | | |
|  | Band-pass filter cutoffs |  | | |
|  | Artefact rejection |  | | |
|  |  |  | | |
| Electrode number | Before the break  Visit date: | | After the break  Visit date: | |
|  | Threshold | AGF slope | Threshold | AGF slope |
| 1 |  |  |  |  |
| 2 |  |  |  |  |
| 3 |  |  |  |  |
| 4 |  |  |  |  |
| 5 |  |  |  |  |
| 6 |  |  |  |  |
| 7 |  |  |  |  |
| 8 |  |  |  |  |
| 9 |  |  |  |  |
| 10 |  |  |  |  |
| 11 |  |  |  |  |
| 12 |  |  |  |  |
| 13 |  |  |  |  |
| 14 |  |  |  |  |
| 15 |  |  |  |  |
| 16 |  |  |  |  |
| 17 |  |  |  |  |
| 18 |  |  |  |  |
| 19 |  |  |  |  |
| 20 |  |  |  |  |
| 21 |  |  |  |  |
| 22 |  |  |  |  |

**eSRT**

| Duration and dates of the break |  | | |
| --- | --- | --- | --- |
| Keep these settings constant throughout testing | Mode of stimulation |  | |
|  | Stimulation rate |  | |
|  | Number of pulse trains |  | |
|  | Starting level |  | |
|  | Final level |  | |
|  | Step size for ascending run |  | |
|  | Step size for descending run |  | |
| Electrode number | Before the break  Visit date: | | After the break  Visit date: |
|  | Threshold | | Threshold |
| 1 |  | |  |
| 2 |  | |  |
| 3 |  | |  |
| 4 |  | |  |
| 5 |  | |  |
| 6 |  | |  |
| 7 |  | |  |
| 8 |  | |  |
| 9 |  | |  |
| 10 |  | |  |
| 11 |  | |  |
| 12 |  | |  |
| 13 |  | |  |
| 14 |  | |  |
| 15 |  | |  |
| 16 |  | |  |
| 17 |  | |  |
| 18 |  | |  |
| 19 |  | |  |
| 20 |  | |  |
| 21 |  | |  |
| 22 |  | |  |

1. Lam C, Moore BCJ, Salorio-Corbetto M, Vickers DA. The relationship between hearing experiences, music-listening behaviors and chord-discrimination abilities for cochlear-implant users. *Trends in Hearing*. 2022;(26):1-18.

2. Gajadeera EA, Galvin KL, Dowell RC, Busby PA. The change in electrical stimulation levels during 24 months postimplantation for a large cohort of adults using the Nucleus® cochlear implant. *Ear and Hearing*. 2017;38(3).
