# Supplementary material for: Effect of Taking a Break From Cochlear-Implant Use for Resolving Facial-Nerve Stimulation: A Case Series
Source: Otol Neurotol. 2025 Dec 22;47(2):e193–200. doi: 10.1097/MAO.0000000000004698 (PMC12777617; doi:10.1097/MAO.0000000000004698)
Supplement: SUPPLEMENTARY MATERIAL [file mao-47-e193-s003.docx]

**Supplemental Digital Content Figures and Tables**

**Supplemental figures**


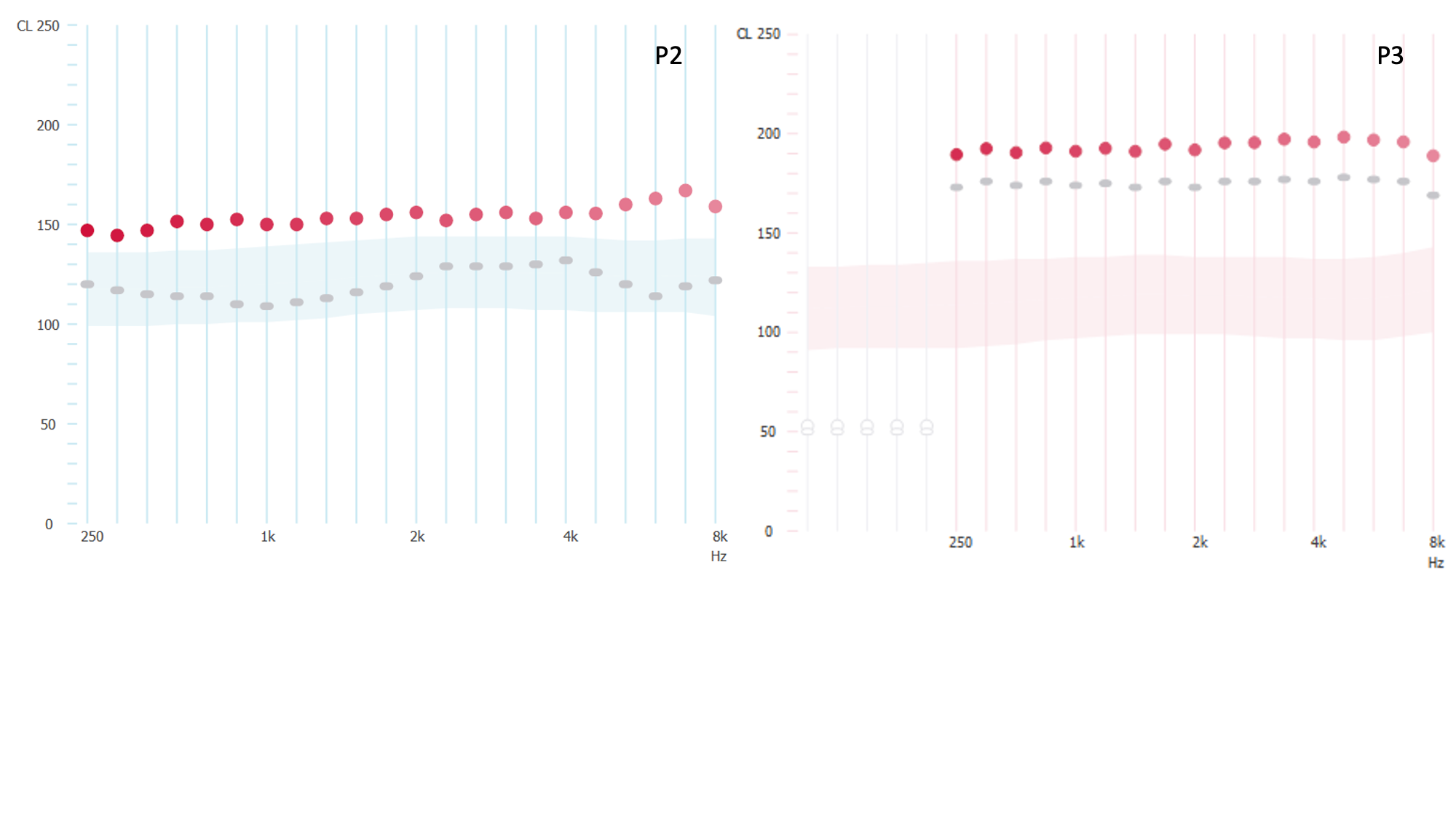


Supp. Fig. 1: Clinical maps at referral for *Patients 2* and *3* (P2 and P3), who had Cochlear^TM^ devices. Data are not shown for *Patient 4*, as no comparison data are available for their device. The shaded area shows the range of upper stimulation levels for 68% of the population^34^.


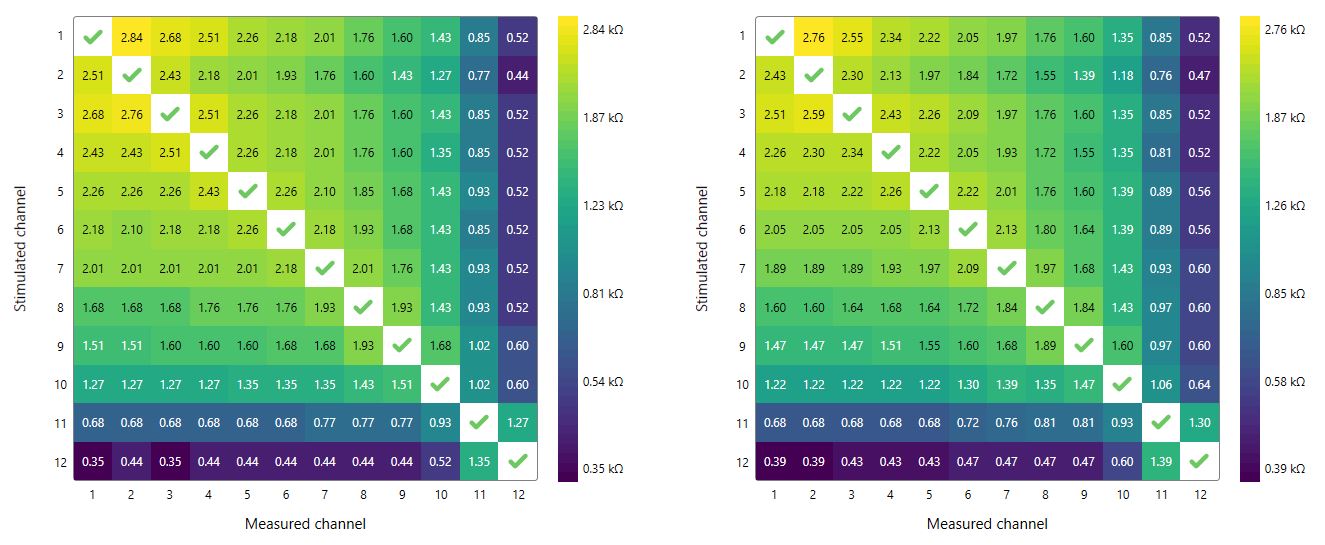


Supp. Fig. 2. MED-EL heat maps representing voltage matrices for *Patient 1*. The heat map on the left was obtained before the break. The heat map on the right was obtained after the break. There were no clear changes in the voltage distribution that could account for a change in FNS patterns.


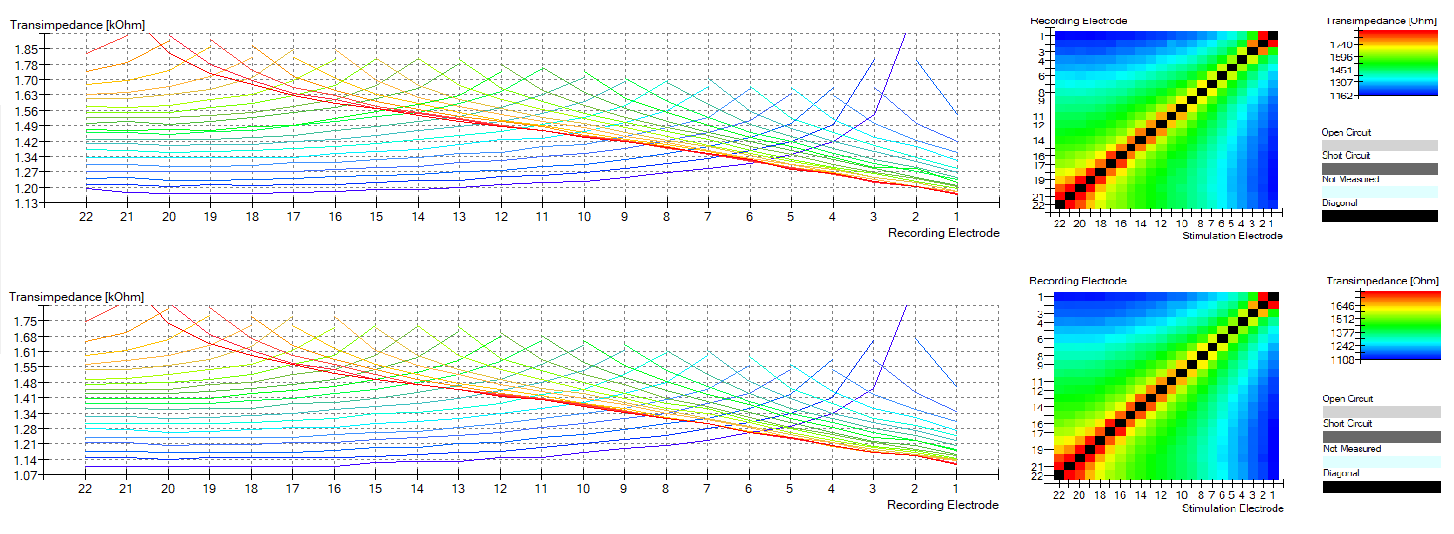


Supp. Fig. 3. Cochlear^TM^ transimpedance matrices for *Patient 2*. The matrix at the top was obtained before the break. The matrix at the bottom was obtained after the break. There were no clear changes in the voltage distribution that could account for a change in FNS patterns.

**Supplementary Tables**

Suppl. Table 1. Clinical map at referral for *Patient 1* (P1), who had a MED-EL device. For reference, MCLs (most comfortable levels) are expected to be between 10 and 40 qu (charge units), with a median around 20 qu; the default value for Thr (threshold) charge is 8% the MCL (Gemma Crundwell, personal communication, 16^th^ October 2023). PD stands for pulse duration.

| Electrode number | 1 | 2 | 3 | 4 | 5 | 6 | 7 | 8 | 9 | 10 |
| --- | --- | --- | --- | --- | --- | --- | --- | --- | --- | --- |
| THR, qu | 15.15 | 16.58 | 15.18 | 17.55 | 15.32 | 16.30 | 16.02 | 17.13 | 19.36 | 25.77 |
| MCL, qu | 40.14 | 39.60 | 41.10 | 42.80 | 39.60 | 44.58 | 43.28 | 40.79 | 44.58 | 48.51 |
| PD (µs) | 42.50 | 42.50 | 42.50 | 42.50 | 34.17 | 42.50 | 42.50 | 34.17 | 42.50 | 42.50 |

Suppl. Table 2. Map parameters for the stimulation levels given in Fig. 1 and Fig. 3. SO = switch-on, 2P = second programming, 1W = one-week programming, 1M = one-months’ programming, 2M = two-months’ programming, 3M = three-months’ months programming, 1Y = one-year programming, Hist. = historical, MP = monopolar, BP = bipolar, ACE = advanced combination encoder, FS4 = fine structure, 4HDCIS = high-definition continuous interleaved sampling, SPEAK = speech peak.

| Patient | Device brand | Time point | Pulse duration (µs) | Pulse rate (pps) | Mode of Stimulation | Strategy | Number of active electrodes |
| --- | --- | --- | --- | --- | --- | --- | --- |
| 1 | MED-EL CONCERTO FLEX28 | SO | 37.08-64.58 (mode 46.25) | 872 | MP | HDCIS | 11/12 |
|  |  | 2P | 27.08-53.75 (mode 40.42) | 1288 | MP | HDCIS | 10/12 |
|  |  | 1W | 27.08-53.75 (mode 40.42) | 1288 | MP | HDCIS | 10/12 |
|  |  | 2M | 40.42-70.42 (mode 50.42) | 819 | MP | HDCIS | 10/12 |
|  |  | 1 year | 45.42-67.92 (mode 56.67) | 2000 | MP | HDCIS | 10/12 |
|  |  | Pre-break | 75.42-150.40 (mode 75.42) | 518 | MP | HDCIS | 10/12 |
|  |  | Post-break | 28.75-50 (mode 28.75) | 1205 | MP | FS4 | 10/12 |
|  |  | 3 months post-break | 25.42-50.42 (mode 31.67) | 1274 | MP | FS4 | 10/12 |
|  |  | 6-months post-break | 50.42 | 750 | MP | FS4 | 10/12 |
|  |  | 12-months post-break | 50 | 750 | MP | FS4 | 9/12 |
|  |  | 40-months post-break | 50 | 750 | MP | FS4 | 9/12 |
| 2 | Cochlear^TM^ CI422 | SO | 37 | 900 | MP1+2 | ACE | 15/22 |
|  |  | 2P | 50 | 900 | MP1+2 | ACE | 22/22 |
|  |  | 1W | 50 | 900 | MP1+2 | ACE | 22/22 |
|  |  | 2M | 50 | 900 | MP1+2 | ACE | 22/22 |
|  |  | 1Y | 50 | 900 | MP1+2 | ACE | 22/22 |
|  |  | Pre-break | 100 | 500 | MP1+2 | ACE | 22/22 |
|  |  | Post-Break | 50 | 250 | MP1+2 | ACE | 22/22 |
|  |  | 1 month post-break | 50 | 250 | MP1+2 | ACE | 22/22 |
|  |  | 11 months post-break | 50 | 250 | MP1+2 | ACE | 22/22 |
|  |  | 17 months post-break | 100 | 500 | MP1+2 | ACE | 22/22 |
|  |  | 42 months post-break | 100 | 500 | MP1+2 | ACE | 22/22 |
| 3 | Cochlear^TM^  CI24RE(CA) | 2P | 50 | 900 | MP1+2 | ACE | 22/22 |
|  |  | 1W | 50 | 900 | MP1+2 | ACE | 22/22 |
|  |  | 1M | 50 | 900 | MP1+2 | ACE | 17/22 |
|  |  | 3M | 50 | 900 | MP1+2 | ACE | 17/22 |
|  |  | 1Y | 88 | 500 | MP2 | ACE | 17/22 |
|  |  | Pre-break | 88 | 500 | MP2 | ACE | 10/22 |
|  |  | Post-break | 88 | 500 | MP2 | ACE | 17/22 |
|  |  | 10 months post-break | 88 | 500 | MP2 | ACE | 17/22 |
|  |  | 38 months post-break | 88 | 500 | MP2 | ACE | 17/22 |
|  |  | 41 months post-break | 88 | 500 | MP2 | ACE | 17/22 |
|  |  | 45 months post-break | 88 | 500 | MP2 | ACE | 17/22 |
|  |  | 49 months post-break | 88 | 500 | MP2 | ACE | 17/22 |
|  |  | 142 months post-break | 100 | 500 | MP2 | ACE | 17/22 |
|  |  | 145 months post-break | 100 | 900 | MP2 | ACE | 17/22 |
| 4 | Cochlear^TM^ CI22M | Hist | 150 | 245-285 | BP+3 | SPEAK | 18/22 |
|  |  | Pre-break | 150 | 491-571 | BP+3 | SPEAK | 11/22 |

Suppl. Table 3. Formulae used for converting clinical units (CU) to charge for Cochlear^TM^ devices (Birgit Philips and Bas van Dijk, personal communication, 18^th^ September and 2^nd^ October 2023).

| Model | Chip | Current in microamps (µa) | Charge in nanocoulombs (nC) |
| --- | --- | --- | --- |
| CI22M | ESL | 1.76*(0.9815+clinical units/30000)^(239-clinical units)*1000 | (µA*10^-6)*(µs*10^-6)*10^9 |
| CI24RE(CA) | CIC4 | 17.5*100(clinical units/255) |  |
| CI422 |  |  |  |

Suppl. Table 4: Changes in lower and upper stimulation levels expressed as percentage of the pre-break dynamic range. Dynamic range (DR), lower stimulation levels (LSL), and upper stimulation levels (USL) are expressed in nC.

| Patient | Pre-Break DR | Mean Pre-Break LSL | Mean Post-Break LSL | LSL difference | LSL change (%) | Mean Pre-Break USL | Mean Post-Break USL | USL difference | USL change (%) |
| --- | --- | --- | --- | --- | --- | --- | --- | --- | --- |
| 1 | 20.07 | 34.26 | 9.97 | 24.29 | 121.02 | 54.33 | 19.65 | 34.68 | 172.80 |
| 2 | 12.83 | 15.44 | 8.87 | 6.57 | 51.21 | 28.27 | 15.37 | 12.90 | 100.55 |
| 3 | 10.73 | 49.02 | 35.86 | 13.16 | 122.68 | 59.75 | 51.30 | 8.45 | 78.75 |

**Appendix 1 –** Sound Diary (separate file)

**Appendix 2 –** Template for monitoring patients taking a break from cochlear-implant stimulation due to FNS (separate file)
